# Agroecosystems shape population genetic structure of the greenhouse whitefly in Northern and Southern Europe

**DOI:** 10.1186/s12862-014-0165-4

**Published:** 2014-07-29

**Authors:** Irina Ovčarenko, Despoina Evripidis Kapantaidaki, Leena Lindström, Nathalie Gauthier, Anastasia Tsagkarakou, Karelyn Emily Knott, Irene Vänninen

**Affiliations:** 1 Department of Biological and Environmental Science, University of Jyvaskyla, Jyvaskyla, FI-40014, Finland; 2 MTT Agrifood Research Finland, Plant Production Research, Tietotie, Animale building, Jokioinen, FI-31600, Finland; 3 Hellenic Agricultural Organization – “Demeter”, Plant Protection Institute of Heraklion, Laboratory of Entomology and Agricultural Zoology, Heraklion, 71003, Greece; 4 Department of Environmental and Natural Resources, University of Patras, Agrinio, Greece; 5 IRD, UMR (INRA, IRD, CIRAD, SupAgro) Centre de Biologie pour la Gestion des Populations (CBGP), Campus international de Baillarguet, Montferrier-sur-Lez cedex, F-34988, France

**Keywords:** Trialeurodes vaporariorum, Pest management, Microsatellite markers, Climate zone, Host adaptation

## Abstract

**Background:**

To predict further invasions of pests it is important to understand what factors contribute to the genetic structure of their populations. Cosmopolitan pest species are ideal for studying how different agroecosystems affect population genetic structure within a species at different climatic extremes. We undertook the first population genetic study of the greenhouse whitefly (*Trialeurodes vaporariorum)*, a cosmopolitan invasive herbivore, and examined the genetic structure of this species in Northern and Southern Europe. In Finland, cold temperatures limit whiteflies to greenhouses and prevent them from overwintering in nature, and in Greece, milder temperatures allow whiteflies to inhabit both fields and greenhouses year round, providing a greater potential for connectivity among populations. Using nine microsatellite markers, we genotyped 1274 *T. vaporariorum* females collected from 18 greenhouses in Finland and eight greenhouses as well as eight fields in Greece.

**Results:**

Populations from Finland were less diverse than those from Greece, suggesting that Greek populations are larger and subjected to fewer bottlenecks. Moreover, there was significant population genetic structure in both countries that was explained by different factors. Habitat (field vs. greenhouse) together with longitude explained genetic structure in Greece, whereas in Finland, genetic structure was explained by host plant species. Furthermore, there was no temporal genetic structure among populations in Finland, suggesting that year-round populations are able to persist in greenhouses.

**Conclusions:**

Taken together our results show that greenhouse agroecosystems can limit gene flow among populations in both climate zones. Fragmented populations in greenhouses could allow for efficient pest management. However, pest persistence in both climate zones, coupled with increasing opportunities for naturalization in temperate latitudes due to climate change, highlight challenges for the management of cosmopolitan pests in Northern and Southern Europe.

## Background

The dispersal of phytophagous insect pests can be enhanced by worldwide trade and human movement [1],[2]. In addition, climate change facilitates movement of various taxa polewards [3],[4]. Following introduction to new habitats, the establishment of insect pest populations can be favored by benign climates, as well as by monocultures in agroecosystems, i.e. agricultural fields and greenhouses [5]-[7]. Low genetic diversity of insect pest populations in newly occupied habitats suggests that even a single successful founder event is enough to establish populations [8],[9]. However, further spread of introduced pests into natural ecosystems depends on the environment surrounding the initial introduction and on the origin of the introduced species [10],[11]. For example, pests of tropical origin may be more likely to establish themselves in the Mediterranean than in the boreal climate zone [12],[13]. At southern latitudes there are suitable climatic conditions and year-round availability of host plants, in both natural ecosystems and densely aggregated agroecosystems. In contrast, at northern latitudes, natural habitats are only seasonally available and greenhouses are often sparsely distributed. Thus, the extent of establishment and spread of invasive pests in the North might be more dependent on the distribution of agroecosystems, particularly greenhouses, than it is in the South.

Enclosed greenhouse environments are designed to reduce evaporation, pest entry [14] and loss of expensive biological pest control agents [15], to ensure efficient crop maintenance. Because greenhouses are relatively closed environments, pest populations in greenhouses might be generally more affected by insecticide applications and host plant changes than are pest populations in fields. These crop management practices can lead to reductions in population size and selection for resistant genotypes in the pests leading to increased homozygosity within and differentiation between pest populations [16]. Thus, populations of insects inhabiting greenhouses might show more genetic differentiation than those in fields. Indeed, populations of phytophagous pests inhabiting greenhouses often show population genetic structure, e.g. *Tetranychus urticae* Koch [17],[18], although dispersal and gene flow can also be restricted among pest populations inhabiting fields, e.g. *Leptinotarsa decemlineata* Say [8].

The greenhouse whitefly (*Trialeurodes vaporariorum* Westwood) is an invasive pest which was brought to Europe (UK) on Orchidaceae from Mexico in 1856 [19]. Soon after introduction, it spread to the European continent, and in 1920 it was recorded in greenhouses in Finland [20]. In Finland, it spread through transportation on plant seedlings above the Arctic Circle to Rovaniemi, bringing considerable damage to tomato and cucumber crops, as well as to ornamental plants [21],[22]. *T. vaporariorum* was reported from the Mediterranean region only later, in 1963 [19]. The species was noticed in Greece (Crete) only in 1978, when it began to cause pest management problems due to its resistance to insecticides [23],[24].

*T. vaporariorum* currently has an almost cosmopolitan distribution [25],[26]. Its success can be attributed to the worldwide distribution of greenhouse habitats, polyphagy [27], its tolerance of higher or lower temperatures than its biological control agents [17],[26], and its haplodiploid mode of reproduction [28]. However, the absence of an overwintering resting stage [29] potentially limits its spread to natural ecosystems. Since the development of *T. vaporariorum* ceases at 8.3°C [30], year-round populations might persist at southern latitudes, where some host plants are available during winter, but are not likely to persist at northern latitudes, where crop cultivation in fields is seasonal and wild host plants decay during winter.

To date, population genetic structure has been analyzed in only a few whitefly species, and little is known about population genetic structure in *T. vaporariorum*. The related *Bemisia tabaci* species complex, particularly Mediterranean *B. tabaci* (Med), is characterized by high genetic diversity and differentiation of populations, as indicated by both mitochondrial and microsatellite markers [31],[32] (except in recently introduced populations in Taiwan and France [33],[34]). Populations of *B. tabaci* (Med) in Greece separated by just a few kilometers show population genetic structure, possibly due to separate founder events or an older population history in this country [35]. Unlike the *B. tabaci* species complex, *T. vaporariorum* populations have low genetic diversity in mitochondrial genes [36],[37]. Recent findings indicate that sequences of three mitochondrial genes and composition of endosymbiont communities from populations sampled from different continents show little variation (Kapantaidaki et al., unpubl.). Analysis of a few nuclear genes (allozymes) in *T. vaporariorum* populations from greenhouses in South Korea revealed their subdivision possibly due to restricted gene flow by natural geographic barriers [38]. However, studies of population genetic structure in *T. vaporariorum* with other, more polymorphic genetic markers, allowing description of more recent evolutionary processes, have not been performed until now. Recent findings of variation in phenotypic responses, particularly diverse responses to insecticide treatments among geographically close populations, suggest differentiation and low gene flow among invaded greenhouses [39].

To understand how insect pests respond to different environmental conditions, such as climate, habitat, and crop management practices, and the role of agroecosystems in shaping population genetic structure, comparative studies of population genetic diversity of pests in different climate zones are necessary. In this study we present the first extensive genetic data on population structure of the greenhouse whitefly. We compare the genetic structure of *T. vaporariorum* populations in Finland and in Greece, representing boreal and Mediterranean climate zones, and evaluate the influence of host plants and agricultural practices on the spatial and temporal population genetic structure of this invasive species. We hypothesize that *T. vaporariorum* populations in Northern Europe are more likely to be genetically differentiated than populations in Southern Europe, because this species is expected to be restricted to greenhouses in the North.

## Methods

### Sampling

**Figure 1:**
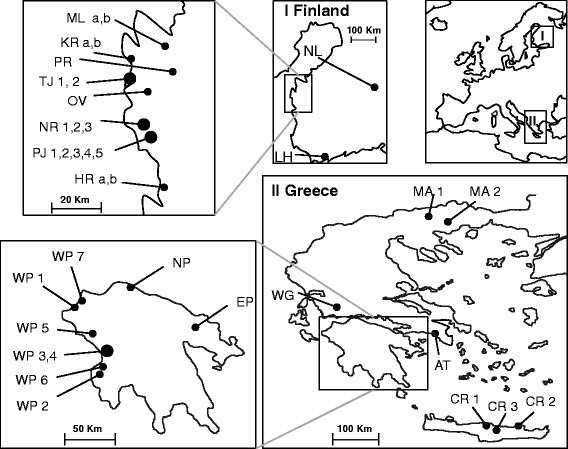
**Maps of sampling locations.** Sample codes are listed in Table 1. I - Finland, II - Greece.

In Finland we sampled commercial greenhouses that operate year-round and produce primarily tomato and cucumber crops of various cultivars (Table 1). Samples were collected from greenhouses belonging to different growers. In total 18 greenhouses were sampled in spring of 2010–2012. Ten of these were sampled twice: in 2010 and in 2011. Sampling was concentrated in Ostrobothnia (16 greenhouses) but also included two distant locations in other parts of the country (Figure 1-I). Ostrobothnia was the focus of our study because in this area we could find multiple greenhouses with different management practices in terms of host plant species, their cultivars (Table 1) and the origin of seedlings. Two to five greenhouses belonging to different growers were sampled within Närpes, Töjby and Pjelax villages. The minimum and maximum distances between these villages were 9 and 32 km, respectively, measured as straight line distance between coordinates. The distances between greenhouses within villages ranged from 1.1 to 3.7 km in Närpes, 0.4 km in Töjby and from 0.28 to 0.9 km in Pjelax.

**Table 1:** Description of the samples collected in Finland and Greece

**Geographical information**					**Host plant**			**Collection**	
			**Geographical coordinates:**						**Date:**
**Country/Region**	**Locality**	**Sample code**	**Latitude**	**Longitude**	**Species**	**Cultivar**	**Family**	**Habitat**	**Month-year**
**Finland**									
Ostrobothnia	Härkmeri	HR a	62.165219	21.467372	Cucumber ^1^	Imea	Cucurbitaceae	G	May-10
		HR b			Tomato ^1^	Espero	Solanaceae	G	Apr-11
	Korsnäs	KR a	62.778983	21.204792	Cucumber	Cadense R2	Cucurbitaceae	G	May-10
		KR b			Cucumber	Cadense R2	Cucurbitaceae	G	Apr-11
	Malax	ML a	62.938797	21.526186	Cucumber ^1^	Diligare	Cucurbitaceae	G	May-10
		ML b			Tomato ^1^	DRW	Solanaceae	G	Apr-11
	Närpes	NR 1a	62.476119	21.416114	Tomato	Encore	Solanaceae	G	May-10
		NR 1b			Tomato	Encore	Solanaceae	G	Apr-11
		NR 2	62.479328	21.395703	Cucumber	Imea	Cucurbitaceae	G	May-10
		NR 3a	62.467842	21.346608	Cherry tomato	Gonchita	Solanaceae	G	May-10
		NR 3b			Tomato	Gonchita	Solanaceae	G	Apr-11
	Pjelax	PJ 1a	62.393006	21.382206	Tomato	Encore	Solanaceae	G	May-10
		PJ 1b			Tomato	Encore	Solanaceae	G	Apr-11
		PJ 2	62.395511	21.381911	Tomato	Encore	Solanaceae	G	May-10
		PJ 3a	62.396372	21.382139	Tomato	Encore	Solanaceae	G	May-10
		PJ 3b			Tomato	Encore	Solanaceae	G	Apr-11
		PJ 4	62.397450	21.375103	Tomato	Dometica	Solanaceae	G	Apr-11
		PJ 5	62.389081	21.371075	Tomato	Dometica	Solanaceae	G	Apr-11
	Pörtom	PR a	62.710939	21.623539	Tomato	Encore	Solanaceae	G	May-10
		PR b			Tomato	Encore	Solanaceae	G	Apr-11
	Töjby	TJ 1a	62.664411	21.221228	Cucumber	Ventura	Cucurbitaceae	G	May-10
		TJ 1b			Cucumber	Logica	Cucurbitaceae	G	Apr-11
		TJ 2a	62.661847	21.226625	Cucumber	Annica	Cucurbitaceae	G	May-10
		TJ 2b			Cucumber	Annica	Cucurbitaceae	G	Apr-11
	Övermark	OV	62.611700	21.471772	Tomato	Several cultivars ^4^	Solanaceae	G	Apr-11
Uusimaa	Lohja	LH	60.176453	23.981306	Cucumber	Imea	Cucurbitaceae	G	Apr-11
Northern Savonia	Nilsiä	NL	63.151436	27.987397	Cucumber ^2^	Imea	Cucurbitaceae	G	Jul-12
**Greece**									
West Peloponnese	Kourtessi	WP 1	37.966667	21.330278	Cucumber	-	Cucurbitaceae	F	Jun-04
	Filiatra	WP 2	37.119983	21.584281	Zuccini	-	Cucurbitaceae	F	Jul-04
	Elea	WP 3	37.372628	21.688894	Eggplant	-	Solanaceae	F	Aug-11
	Prasidaki	WP 4	37.397167	21.711822	Bean	-	Fabaceae	F	Aug-11
	Anemochori	WP 5	37.588725	21.538794	Tomato	-	Solanaceae	F	Sep-11
	Terpsithea	WP 6	37.227417	21.628542	Bean	-	Fabaceae	F	Sep-11
	Andravida	WP 7	38.007222	21.395833	Marrow	-	Cucurbitaceae	F	Sep-11
North Peloponnese	Aigio	NP	38.216853	22.114178	Rose	-	Rosaceae	G	Aug-11
West Greece	Agrinio	WG	38.579722	21.418056	Tomato	-	Solanaceae	G	Jun-11
East Peloponnese	Nafplion	EP	37.745556	22.850278	Bean	-	Fabaceae	F	Oct-11
Attica	Athens	AT	37.983147	23.706583	Eggplant	-	Solanaceae	G	Apr-05
Island of Crete	Fodele	CR 1	35.398228	24.963689	Rose	-	Rosaceae	G	Mar-10
	Sissi	CR 2	35.305961	25.535006	Rose	-	Rosaceae	G	Apr-11
	Malades	CR 3	35.268528	25.104956	Datura	-	Solanaceae	G	Apr-11
Macedonia	Serres	MA 1	41.225933	23.361469	Tomato	-	Solanaceae	G	May-11
	Drama	MA 2	41.124744	24.162803	Sweet pepper ^3^	-	Solanaceae	G	May-11

Lower case letters adjacent to population codes indicate the same location sampled in 2010 and 2011.

G indicates samples collected from greenhouses, F – from fields.

^1^Cucumber and tomato were growing in the same greenhouse compartment.

^2^Cucumber and tomato were growing in different greenhouse compartments.

^3^Tomato and eggplant were growing in the same greenhouse compartment.

^4^Encore, Careza, Dometica, Dirk and Axxion cultivars grown in the same greenhouse compartment.

In Greece we sampled 16 agroecosystems in different seasons over several years: 2004–2011. These included eight greenhouses and eight fields growing various crop plants (Table 1) which were distributed throughout mainland Greece, the Peloponnese and the Island of Crete (Figure 1-II). Sampling was concentrated in the fields of West Peloponnese because open environments in this region cover the expected range (7–20 km) of the potential dispersal abilities of whiteflies (natural or by wind, as known from the *B. tabaci* species complex; [40],[41]). In West Peloponnese, the minimum distance between samples ranged from 3.4 km (between WP3 and WP4) to 24.4 km (between WP3 and WP5), and the maximum distance between locations reached 100 km.

During sampling, both genders of whiteflies were collected using a mouth aspirator. The whiteflies were preserved in 90% Ethanol and stored at 4°C until they were sexed and used for genotyping. Since *T. vaporariorum* is a haplodiploid species, only adult females were chosen for genotyping.

### DNA extraction and microsatellite genotyping

Total genomic DNA was extracted from each individual female as described in Tsagkarakou et al. [42]. Nine microsatellite markers (Table 2) out of the 13 characterized in Molecular Ecology Resources Primer Development Consortium et al. [43] were used to genotype 1274 *T. vaporariorum* females, 800 from Finland and 474 from Greece (Table 3). Four of the microsatellite markers described previously did not amplify consistently, and thus, were excluded from this study. Three multiplex amplification reactions were performed as described in Molecular Ecology Resources Primer Development Consortium et al. [43] with slight modifications. Diluted amplification products were separated on an ABI 3130xl genetic analyzer (Applied Biosystems). Allele sizes were scored against GeneScan™ 500 LIZ standard using GeneMapper® v 4.0 software (both Applied Biosystems) and were confirmed manually.

**Table 2:** **Characteristics of the nine polymorphic microsatellite loci analysed in** ***T. vaporariorum***

**Locus (Genbank Accession no.)**	**Primer sequence (5′-3′) (F: [dye]-forward; R: reverse)**	**Repeat motif (cloned allele)**	**Size range (bp)**	**No. of alleles**	***H***_**0**_**/*H***_**E**_**Finland**	***H***_**0**_**/*H***_**E**_**Greece**
Tvap-1-1C	F: [6-FAM]- GAGACTCCACGATGTCTGTC	(GT)_6_ GG(GT)_9_	195-215	3	0.469/0.499*	0.455/0.461
(GF112015)	R: TTCCCCTATCGTATGTTCAC					
Tvap-1-2	F: [VIC]- CTGTGAATCCCTCAGAAATC	(GT)_6_	233-236	2	0.094/0.108	0.299/0.308
(GF112025)	R: TGACCTCTCTCAGGCTTTTA					
Tvap-3-1	F: [PET]- GAGATGGACAAACTACAACG	(AC)_15_	228-230	2	0.246/0.437*	0.268/0.355*
(GF112016)	R: GATTGGATGTCGTGGTTG					
Tvap-3-2	F: [6-FAM]- GGAGGTCATTACTCATTTCG	(AC)_6_	170-182	4	0.401/0.405	0.522/0.581
(GF112017)	R: CATAAATTTTCGGCTCACTC					
Tvap-3-3	F: [VIC]- CGCAAATCATACTTCCTTTC	(CA)_5_	235-237	2	0.417/0.412	0.496/0.459
(GF112019)	R: AAATACAGGCGACTCATGTC					
Tvap-4-2	F: [NED]- GGTGGTATTGTGGCGTC	(GA)_29_	298-314	7	0.446/0.468	**0.585/0.667**
(GF112027)	R: CTGCCTCTTATGACTCTTCC					
Tvap-1-4	F: [PET]- GATTTAGCCCAGTTCATTTG	(TG)_5_	265-267	2	0.091/0.097	0.137/0.179*
(GF112020)	R: CTTCAGTTGAGCTGCTGATG					
Tvap-1-5	F: [6-FAM]- CAGTTGTGGTAGTGTGGTG	(TG)_12_	124-146	10	0.416/0.411	**0.703/0.757**
(GF112028)	R: CTCATCGGCTCATACATTC					
Tvap-2-2C	F: [VIC]- CTGAAAGTCTTATTAGAGCC	(TC)_8_ GC (TC)_10_	210-220	6	0.568/0.55	0.588/0.608
(GF112021)	R: CTAACTGATTCCATAGTCG					

No. of alleles indicates the maximum number of alleles found in this study.

*H*_O_, observed heterozygosity; *H*_E_, unbiased expected heterozygosity.

*indicate *H*_*O*_*/H*_*E*_ values with potential presence of null alleles with frequency > 0.2.

***H***_***O***_***/H***_***E***_ in bold indicate loci with significant deviations from Hardy–Weinberg equilibrium in terms of heterozygote deficiency after Bonferroni correction (no significant heterozygote excess was detected).

Heterozygosities, deviations from HWE and null allele frequencies were estimated over 800 females from 18 samples in Finland and 474 females from 16 samples in Greece.

**Table 3:** **Genetic diversity estimated over the nine microsatellite loci for samples of** ***T. vaporariorum***

**Country**	**Region**	**Locality**	**Sample code**	***N***	***N***_**A**_**(±SE)**	***H***_***O***_ ***/H***_***E***_
**Finland**	Ostrobothnia	Härkmeri	HR a,b	30 + 30	2.556(±0.294)	0.375/0.435*
		Korsnäs	KR a,b	29 + 30	2.333(±0.373)	0.222/0.254*
		Malax	ML a,b	30 + 30	2.667(±0.236)	0.375/0.404
		Närpes	NR 1a,b	30 + 30	3.111(±0.423)	0.369/0.406
			NR 2	30	2.333(±0.167)	0.256/0.341*
			NR 3a,b	30 + 30	2.889(±0.351)	0.308/0.375*
		Pjelax	PJ 1a,b	30 + 30	2.889(±0.389)	0.375/0.429*
			PJ 2	30	2.667(±0.236)	0.422/0.422
			PJ 3a,b	30 + 30	3.111(±0.455)	0.396/0.429*
			PJ 4	30	2.667(±0.289)	0.359/0.417*
			PJ 5	30	2.667(±0.333)	0.407/0.406
		Pörtom	PR a,b	30 + 30	3.444 (±0.669)	0.434/0.484
		Töjby	TJ 1a,b	30 + 30	3.222 (±0.494)	0.570/0.556*
			TJ 2a	30	3.333 (±0.577)	0.465/0.517
			TJ 2b	30	3.667 (±0.707)	0.554/0.531*
		Övermark	OV	30	3.556 (±0.648)	0.511/0.543*
	Uusimaa	Lohja	LH	21	3.667 (±0.799)	0.541/0.529
	Northern Savonia	Nilsiä	NL	30	3.333 (±0.645)	0.448/0.512
**Greece**	West Peloponnese	Kourtessi	WP 1	30	3.222(±0.494)	0.422/0.450
		Filiatra	WP 2	29	3.333 (±0.553)	0.415/0.524
		Elea	WP 3	30	3.444 (±0.626)	0.459/0.540
		Prasidaki	WP 4	30	2.667 (±0.236)	0.409/0.457
		Anemochori	WP 5	30	3.444 (±0.603)	0.437/0.394
		Terpsithea	WP 6	30	3.222 (±0.494)	0.428/0.469*
		Andravida	WP 7	30	3.111 (±0.484)	0.441/0.419
	North Peloponnese	Aigio	NP	30	3.222 (±0.494)	0.277/0.396
	West Greece	Agrinio	WG	30	3.444 (±0.689)	**0.395/0.455**
	East Peloponnese	Navplion	EP	30	3.222(±0.494)	**0.422/0.450**
	Attica	Athens	AT	28	3.333 (±0.553)	0.415/0.524*
	Island of Crete	Fodele	CR 1	30	3.444 (±0.626)	0.459/0.540
		Sissi	CR 2	30	2.667 (±0.236)	0.409/0.457
		Malades	CR 3	30	3.444 (±0.603)	0.437/0.394*
	Macedonia	Serres	MA 1	27	3.222 (±0.494)	0.428/0.469*
		Drama	MA 2	30	3.111 (±0.484)	0.441/0.419

For Finland the *combined* dataset, which pooled samples from consecutive years at the same location (except TJ 2) is described, since it was used in the majority of analyses. Lower case letters adjacent to population codes indicate the same location sampled in 2010 and 2011. N number of analyzed females, *H*_*O*_ observed and *H*_*E*_ expected heterozygosity and *N*_*A*_ mean number of alleles per population averaged over 9 loci. * indicate *H*_*O*_*/H*_*E*_ values in samples with null allele frequency > 0.2. ***H***_***O***_***/H***_***E***_ in bold indicate loci with significant deviations from Hardy–Weinberg equilibrium in terms of heterozygote deficiency after Bonferroni correction (no significant heterozygote excess was detected).

### Data analysis

To analyze genetic distance between samples, both between and within countries, pairwise estimates of *F*_*ST*_ were calculated in Arlequin 3.11 [44]. The significance of the genetic distances at the 0.05 level was tested by permuting the individuals or genotypes between the samples 110 times and adjusting *P* values with strict Bonferroni correction. Since all pairwise *F*_*ST*_ between samples from Finland and Greece showed statistically significant differences, and due to the low probability of gene flow between the two distant countries, all further analyses were done for each country separately.

The samples taken in two consecutive years from the same greenhouse in Finland showed no genetic differentiation, except for one sampling location (TJ-2 a and TJ-2 b). Therefore, in most analyses we used a *combined* dataset, which pooled samples collected from the same greenhouse in 2010 and 2011, (except for TJ-2 a and TJ-2 b, which were considered as separate samples). For other analyses (specified below) we used a *separated* dataset, in which each sampling effort in Finland was considered a separate sample. Each sampling effort in Greece was considered a separate sample in all analyses, because none of the locations were sampled in consecutive years.

For each sample, mean observed (*H*_*O*_) and expected heterozygosity (*H*_*E*_), and mean number of alleles (*N*_*A*_) per locus were calculated using GenAlEx v. 6.5 [45]. Observed and expected heterozygosities were also calculated for each locus over the total data from each country. Departure from Hardy-Weinberg expectations (HWE) was tested with 1000 permutations using a global test across loci or samples as implemented in GENEPOP v. 4.2 [46]. The test was performed using Fisher’s method, testing hypotheses of heterozygote deficiency and heterozygote excess [47], and producing global p value estimates for each sample over all loci and for each locus over samples from Finland and Greece. Genotypic linkage disequilibrium for each pair of loci in the samples was tested using the log likelihood ratio statistic (G-test) as implemented in GENEPOP v. 4.2. For multiple tests, statistical significance was adjusted using strict Bonferroni corrections [48]. The samples were analyzed for potential scoring errors in all loci using MICRO-CHECKER v. 2.2.3 and the frequency of null alleles (*f*) was estimated [49].

To investigate the relationship between genetic and geographic distance, isolation by distance was analyzed in GenAlEx v. 6.5 [45]. Genetic distance was defined by pairwise linear *F*_*ST,*_ (*F*_*ST*_/(1- *F*_*ST*_)), and geographic distance was defined as pairwise distances generated from geographical coordinates expressed in decimal degrees. The correlation between the two data matrices was assessed using a Mantel test and its significance estimated by *P* values, the regression coefficient (*R*^*2*^), and the mean correlation coefficient (*R*_*XY*_) over 999 random permutations of linear *F*_*ST*_ values as implemented in GenAlex v.6.5. Isolation by distance was assessed with smaller subsets of the data as well as using the full datasets, to evaluate the influence of scale on the relationships.

Analyses of molecular variance (AMOVA) were performed using Arlequin 3.11 [44] to estimate and compare the percentage of genetic variation explained by different hierarchical groups (i.e. individual, sample, group of samples). Four analyses were constructed to test the following groups: 1) country (Finland vs. Greece), 2) host plant species (cucumber vs. tomato) in samples from Finland only, 3) host plant botanical family (Cucurbitaceae vs. Solanaceae vs. Fabaceae vs. Rosaceae) in samples from Greece only, and 4) habitat (greenhouse vs. field) in samples from Greece only. For analysis 2, samples HR, ML and NL were excluded since the whiteflies in these greenhouses might have been exposed to both cucumber and tomato grown in the same compartment or greenhouse. However, in analysis 3, sample MA 2 was not excluded because several hosts grown in the same compartment belonged to the same family (Solanaceae) (see Table 1).

To assess the level of genetic differentiation between groups defined above for AMOVA, we compared summary statistics calculated for the different groups: *F*_*IS*_ (inbreeding coefficient measuring heterozygote deficit within populations), *F*_*ST*_ (a measure of population structure and heterozygote deficit among populations), allelic richness (measure of the number of alleles independent of sample size), *H*_E_ (unbiased expected heterozygosity) and *H*_O_ (observed heterozygosity). FSTAT v. 2.9.3 [50] was used to calculate the average (over samples and loci; weighted by sample size) of the chosen statistics for each group and for their comparison. Statistical significance was assessed after 1000 permutations. As in the AMOVA groups, some samples were excluded because multiple hosts were grown in the same compartment or greenhouse (see above).

The relationship between environmental variables and genetic structure of the studied populations was estimated using default settings of the software GESTE v. 2.0 [51]. The software gives the highest posterior probability (*Pr*) to the model explaining genetic structure the best, evaluating environmental variables separately and in combination through a generalized linear model. In this analysis, the *separated* data set for the samples from Finland was used. Latitude, host plant species, cultivar, crop source and year of sampling were evaluated as explanatory variables of population structure in Finland. Latitude, longitude, four host plant families and habitat (field or greenhouse) were evaluated as explanatory variables of population structure in Greece.

Bayesian clustering analysis implemented in STRUCTURE v.2.3.4 [52] was used to infer the number of genetically distinct clusters (*K*) in each country using a model of no admixture, correlated allele frequencies and including the sampling location as a prior [53]. Initial analyses were performed both with admixture and no admixture models, but the later was selected since visualization of the results was more straightforward and no differences in the most likely number of clusters were observed for the two models. Analysis parameters included a burn-in period of 250,000 followed by 500,000 MCMC iterations. For each dataset, Finland and Greece, we tested *K* from 2 to 10, with ten replicate analyses per value of *K*. Subsets of each dataset were analyzed with the same settings. The most likely number of clusters in our samples was determined using the Δ*K* approach [54] as implemented in Structure Harvester v. 0.56.3 [55]. Results were visualized as bar plots by finding the optimal alignment of the ten replicate analyses of the “best” *K* in CLUMPP v. 1.1.2 [56] using the Greedy algorithm and 1000 random input orders, and then by creating graphics in Distruct v. 1.1 [57]. For Finland, the *combined* dataset was used first, and then two subsets of the data were created. In these subsets, the samples were grouped by host plant species and samples HR a, b and ML a, b were separated since they had been collected from different hosts (see Table 1). Samples HR, ML and NL were included in both data subsets since these samples might have been exposed to several hosts grown in the same compartment or greenhouse. For Greece, all samples were first analyzed together, then data subsets were created grouping samples by habitat (field or greenhouse). The definition of the data subsets (by host plant species or habitat) was chosen after considering the results of initial analyses with the full data sets and our analysis with GESTE v. 2.0 [51].

## Results

### Genetic diversity of microsatellite loci and samples

Significant deviations from HWE through heterozygote deficiencies were detected at one locus (Tvap 3–1) and 4 loci (Tvap-4-2, Tvap-1-4, Tvap-1-5 and Tvap-3-1) in the Finnish and Greek samples, respectively (Table 2). At the sample level, a test of HWE across the nine microsatellite loci indicated significant heterozygote deficiency in two Finnish and three Greek samples (Table 3). There were no cases of significant heterozygote excess.

Three loci showed a null allele frequency > 0.2: Tvap-1-1C (2 samples), Tvap-3-1 (12 samples) and Tvap-1-4 (1 sample). For each of these loci, the frequency of null alleles within a sample varied: *f* = 0.110-0.258 (Tvap-1-1C), *f* = 0.164-0.401 (Tvap-3-1), and *f* = 0.166-0.238 (Tvap-1-4), and the average frequency of null alleles over the samples ranged from 0.119 to 0.250. No cases of large allele drop out were found. Even though null alleles are present, deviations from HWE could be also due to significant homozygosity in populations inhabiting the human-mediated environment (i.e. due to population bottlenecks and inbreeding), rather than due to significant genotyping errors.

Genotypic linkage disequilibrium tested for each pair of loci for each sample revealed a potential association between loci Tvap-1-1 and Tvap 3–1 in sample PJ 4. Since locus Tvap-3-1 was characterized by homozygote excess and had a high frequency of null alleles only in sample PJ 4 (*f* = 0.401), we suspect that the linkage disequilibrium indicated for this sample does not reflect a true association between the loci. Therefore, data from all nine loci were used in the analyses.

### Differences between Finland and Greece

AMOVA indicated significant genetic structure between the two geographic areas (Table 4). The percentage of variation explained by country of origin, Finland vs. Greece, was higher than that among the samples within each country (9.90% and 6.87%, respectively; Table 4), indicating that overall genetic variation might be explained by these groups. *T. vaporariorum* from the two countries also differed significantly in their global observed and expected heterozygosities (Finland: *H*_*O*_*/H*_*E*_ = 0.350/0.385 vs. Greece: *H*_*O*_*/H*_*E*_ = 0.451/0.496), and in allelic richness (2.498 vs. 3.234 for Finland vs. Greece, respectively) (all *P* = 0.001). However, *F* statistics calculated for each country did not differ statistically (Finland/Greece; *F*_*IS*_: 0.091/0.090, *P* = 0.157; *F*_*ST*_: 0.093/0.055, *P* = 0.976). Nevertheless, the range of pairwise *F*_*ST*_ values between samples within countries was broader for Finland (−0.006 < *F*_*ST*_ < 0.533) than it was for Greece (−0.007 < *F*_*ST*_ < 0.164) (Tables 5A and B).

**Table 4:** **Distribution of the molecular variance between and within four groups of samples of** ***T. vaporariorum***

**Source of variation**	**d.f.**	**Sum of squares**	**Variance components**	**Percentage of variation**	**Fixation indices**	***P*** **values**
Between countries	1	275.947	0.221	9.900	*F*_*CT*_: 0.099	0 ± 0
Among samples within countries	32	425.048	0.153	6.870	*F*_*SC*_: 0.076	0 ± 0
Within samples	2514	4665.034	1.856	83.240	*F*_*ST*_*:* 0.168	0 ± 0
Between host plant groups in Finland^1^	1	34.818	0.031	1.690	*F*_*CT*_: 0.017	0.036 ± 0.006
Among samples within groups	13	195.663	0.157	8.590	*F*_*SC*_: 0.087	0 ± 0
Within samples	1285	2111.236	1.643	89.720	*F*_*ST*_: 0.103	0 ± 0
Among host plant groups in Greece^2^	3	32.540	0.006	0.250	*F*_*CT*_: 0.002	0.266 ± 0.013
Among samples within groups	12	114.290	0.124	5.330	*F*_*SC*_: 0.053	0 ± 0
Within samples	932	2045.532	2.195	94.420	*F*_*ST*_: 0.056	0 ± 0
Between habitats in Greece	1	32.665	0.052	2.200	*F*_*CT*_: 0.022	0.001 ± 0.001
Among samples within groups	14	114.165	0.010	4.290	*F*_*SC*_: 0.044	0 ± 0
Within samples	932	2045.532	2.195	93.510	*F*_*ST*_: 0.065	0 ± 0

^1^Between groups of samples collected from Cucurbitaceae, Solanaceae host plant families in Finland (samples HR, ML and NL are not included, see Methods for details).

^2^Among groups of samples collected from Cucurbitaceae, Solanaceae, Fabaceae and Rosaceae host plant families in Greece.

**Table 5:** **Pairwise estimates of** ***F***_***ST***_**between samples in Finland (A) and Greece (B) over the nine microsatellite loci**

**AFinland**																		
	**HR**	**KR**	**ML**	**PJ 1**	**PJ 2**	**PJ 3**	**PJ 4**	**PJ 5**	**NR 1**	**NR 2**	**NR 3**	**PR**	**OV**	**TJ 1**	**TJ 2a**	**TJ 2b**	**LH**	**NL**
**HR**	0.000																	
**KR**	0.207	0.000																
**ML**	0.025	0.181	0.000															
**PJ 1**	0.055	0.173	0.024	0.000														
**PJ 2**	0.052	0.222	0.027	**0.009**	0.000													
**PJ 3**	0.058	0.201	0.022	**0.015**	**0.003**	0.000												
**PJ 4**	0.064	0.216	**0.027**	**−0.004**	**−0.006**	**0.002**	0.000											
**PJ 5**	0.073	0.222	0.044	**0.004**	**0.001**	**0.018**	**−0.005**	0.000										
**NR 1**	0.050	0.204	**0.010**	**−0.004**	**0.017**	**0.012**	**0.006**	**0.013**	0.000									
**NR 2**	0.095	0.240	0.044	0.036	0.067	0.065	0.052	0.053	**0.023**	0.000								
**NR 3**	0.079	0.207	**0.024**	**0.029**	0.059	0.051	0.044	0.043	**0.010**	**0.001**	0.000							
**PR**	0.082	0.212	0.053	0.080	0.109	0.010	0.096	0.116	0.063	0.050	0.045	0.000						
**OV**	0.048	0.217	**0.015**	0.024	0.026	0.028	**0.023**	**0.033**	**0.024**	0.039	**0.027**	0.034	0.000					
**TJ 1**	0.113	0.311	0.064	0.077	0.104	0.094	0.088	0.087	0.050	**0.011**	0.021	0.056	0.044	0.000				
**TJ 2a**	0.085	0.263	0.035	0.051	0.070	0.053	0.058	0.061	0.025	**0.003**	**0.001**	0.033	**0.030**	**0.002**	0.000			
**TJ 2b**	0.248	0.533	0.236	0.197	0.225	0.215	0.212	0.206	0.193	0.183	0.211	0.232	0.198	0.129	0.185	0.000		
**LH**	0.131	0.293	0.115	0.063	0.073	0.102	0.057	0.055	0.090	0.083	0.104	0.156	0.078	0.119	0.123	0.196	0.000	
**NL**	0.060	0.327	0.031	0.066	0.089	0.045	0.086	0.111	0.034	0.109	0.077	0.050	**0.035**	0.096	0.061	0.294	0.227	0.000
**BGreece**																		
	**AT**	**CR 1**	**CR 2**	**CR 3**	**WG**	**MA 1**	**MA 2**	**NP**	**WP 1**	**WP 2**	**WP3**	**WP4**	**WP5**	**WP6**	**WP7**	**EP**		
**AT**	0.000																	
**CR 1**	0.144	0.000																
**CR 2**	0.098	0.057	0.000															
**CR 3**	0.154	0.010	0.064	0.000														
**WG**	0.116	0.090	0.028	0.036	0.000													
**MA 1**	0.164	0.037	**0.034**	0.096	0.084	0.000												
**MA 2**	0.140	0.070	0.055	0.116	0.044	0.079	0.000											
**NP**	0.089	0.047	**0.007**	0.084	0.067	**0.015**	0.077	0.000										
**WP 1**	0.102	0.096	0.043	0.081	0.037	0.075	0.099	0.050	0.000									
**WP 2**	0.085	0.097	0.052	0.087	0.027	0.100	0.070	0.071	0.029	0.000								
**WP 3**	0.113	0.097	0.041	0.095	0.045	0.073	0.076	0.051	**0.012**	**0.011**	0.000							
**WP 4**	0.113	0.089	0.051	0.075	0.035	0.076	0.076	0.056	**0.006**	**0.012**	**−0.006**	0.000						
**WP 5**	0.078	0.099	0.042	0.077	**0.028**	0.079	0.078	0.043	**0.003**	**0.004**	**−0.004**	**−0.005**	0.000					
**WP 6**	0.087	0.066	**0.011**	0.056	**0.017**	0.047	0.051	0.026	**0.004**	**0.010**	**−0.001**	**−0.002**	**−0.004**	0.000				
**WP 7**	0.097	0.079	**0.021**	0.054	**0.023**	0.048	0.067	0.025	**0.002**	0.023	**0.004**	**0.002**	**−0.006**	**−0.007**	0.000			
**EP**	0.093	0.085	0.064	0.077	**0.018**	0.083	0.057	0.070	0.045	0.038	0.051	0.032	0.030	**0.029**	0.034	0.000		

### Population structure in Finland

Seventy nine percent of pairwise *F*_*ST*_ comparisons (121 of 154) between samples from Finland showed significant population differentiation (Table 5A). Some populations (HR, KR, PR, TJ 2b and LH) were differentiated from all other samples (Table 5A). However, one of the samples most distant from the Ostrobothnia region (NL) was not significantly different in pairwise *F*_*ST*_ from one of the Ostrobothnian samples (OV). For samples collected from different greenhouses at the same location (NR, PJ and TJ), there was no significant genetic structure, except for TJ: TJ1 was not differentiated from TJ 2a, but both of these samples were differentiated from TJ 2b. Moreover, some of these samples were also not differentiated from other samples in a neighboring village (Table 5A).

There was no evidence for isolation by distance when all Finnish samples were analyzed together (*R*_*XY*_ = 0.259, *R*^*2*^ = 0.067, *P* = 0.190), nor when samples outside Ostrobothnia were excluded from the dataset (*R*_*XY*_ = 0.138, *R*^*2*^ = 0.019, *P* = 0.211). However, it was moderately strong and significant (*R*_*XY*_ = 0.480, *R*^*2*^ = 0.231, *P* = 0.001), when only the core samples in Ostrobothnia were analyzed (outlying samples HR, ML and KR). The data was explained best when only NR, PJ and TJ samples were analyzed (*R*_*XY*_ = 0.618, *R*^*2*^ = 0.382, *P* = 0.001) and at the smallest geographic scale, when including only NR and PJ samples (*R*_*XY*_ = 0.876, *R*^*2*^ = 0.767, *P* = 0.007).

In AMOVA, the percentage of variation explained by host plant species (cucumber vs. tomato) was low (1.69%) but statistically significant (*P* = 0.036). The percentage of variation explained by the groups was lower than that among samples within each group (8.59%, Table 4). However, *T. vaporariorum* collected from cucumber and tomato hosts differed significantly in allelic richness (*P* = 0.005, 2.25 vs.2.61), heterozygosities (*P* = 0.002, *H*_*O*_*/H*_*E*_: 0.278/0.300 vs. 0.371/0.413), and genetic differentiation (*P* = 0.002, *F*_*ST*_: 0.228 vs.0.043) (cucumber vs. tomato respectively). Overall, samples collected from cucumber exhibited less genetic diversity and a higher degree of genetic differentiation than samples collected from tomato.

Furthermore, host plant species was indicated to be the most important factor explaining population genetic structure according to our analysis with GESTE. The model with highest posterior probability (*Pr* = 0.81) was the model with host plant species. All other models including other environmental factors or their combination had very low probability values. For example, *Pr* = 0.0446 was determined for the model including crop origin in combination with host plant species and *Pr* = 0.0443 was determined for the model including crop origin in combination with cultivar, to mention a few.

**Figure 2:**
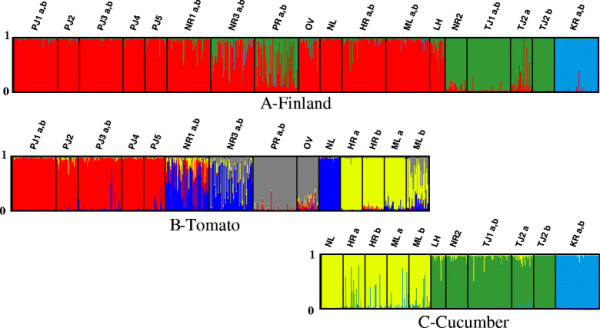
**Genetic clusters of*T. vaporariorum*sampled in Finland delineated by Bayesian analysis implemented in STRUCTURE[**[52]**].** In each graph individuals are represented by vertical bars broken into *K* differently colored genetic clusters, with length proportional to assignment in each cluster. Probability of assignment of each individual is indicated from 0 to 1. Individuals are grouped by sample with the sample code listed at the bottom of the bar graph. **A**: Analysis of all 18 samples, 800 individuals, *K* = 3. **B**: Analysis of a subset of 14 samples collected from tomato, 570 individuals, *K* = 4. **C**: Analysis of a subset of 11 samples collected from cucumber, 380 individuals, *K* = 3. The *combined* dataset was used in the initial run **(A)**. Samples HR, ML and NL were included in analysis of both host plant groups **(B and C)** since these samples might have been exposed to several hosts grown in the same compartment or greenhouse. Samples HR a, b and ML a, b were separated in analyses **B** and **C** as they were exposed to different hosts.

The Bayesian analysis of population structure indicated that the 18 samples from Finland (Table 1) represent three main genetic clusters (*K* = 3; Figure 2A). The genetic clusters could be characterized by both host plant species and geographic location. Most samples were clearly assigned to one of the three clusters (assignment higher than 80%). However, for samples PR and NR3, the assignment was mixed between cluster 1 and 2 (PR: *K*_*1*_ = 0.453 and *K*_*2*_ = 0.517; NR3: *K*_*1*_ = 0.769 and *K*_*2*_ = 0.195). Majority of samples in *K*_*1*_ were collected from tomato, with two exceptions: LH and NL were collected from cucumber and these samples were geographically distant from the other samples in this cluster. *K*_*2*_ included four samples collected from cucumber (NR 2, TJ 1, TJ 2a and TJ 2b). Notably, NR 2 was assigned to a different cluster than the samples from other greenhouses in Närpes. *K*_*3*_ corresponded to a single greenhouse growing cucumber (KR), which, being surrounded by forests was somewhat geographically isolated from other greenhouses in Ostrobothnia.

To resolve sub-clustering, further Bayesian analyses with STRUCTURE were conducted for samples collected from tomato and cucumber hosts separately (Figure 2B and C), since host plant species was the major component of genetic structure revealed in our initial analysis and our analysis with GESTE. Some samples were included in both subsequent runs (see Methods). The subset of samples from tomato (*K*_*1*_ in the initial analysis) was characterized best by four sub-clusters (*K* = 4) (Figure 2B). Whiteflies collected from PJ formed sub-cluster 1 (*K*_*1.1*_). NR 1 and NR 3 were not clearly resolved and partially formed sub-cluster 2 (*K*_*1.2*_) with NL (NR 1a, b: *K*_*1.2*_ = 0.584 and *K*_*1.1*_ = 0.287; NR 3a, b: *K*_*1.2*_ = 0.542 and *K*_*1.3*_ = 0.372). PR and OV formed sub-cluster 3 (*K*_*1.3*_), whereas HR and ML formed sub-cluster 4 (*K*_*1.4*_). Assignment was not resolved well for ML b: *K*_*1.4*_ = 0.543 and *K*_*1.3*_ = 0.297. Additional analysis of an even smaller subset of samples (PJ 1-PJ 5) collected from greenhouses that were all growing tomato and located within the natural dispersal range expected for the whitefly, revealed complete genetic homogeneity (data not shown) and indicated high gene flow at this location. The subset of samples collected from cucumber (*K*_*2*_ and *K*_*3*_ in the initial analysis) was characterized best by three well-resolved sub-clusters (*K* = 3), all with 85-100% assignment (Figure 2C). HR, ML and NL formed sub-cluster 1 (*K*_*2.1*_), whereas LH, NR 2, TJ 1, TJ 2a and TJ 2b formed sub-cluster 2 (*K*_*2.2*_). KR alone formed sub-cluster 3 (*K*_*3.1*_).

### Population structure in Greece

Seventy five per cent of pairwise *F*_*ST*_ comparisons (91 out of 120) between samples from Greece showed significant population differentiation (Table 5B). Samples AT, CR 1, CR 3 and MA 2 differed significantly from all other samples (Table 5B). Significant differentiation among samples was absent only in some geographically close sites, in particular among the samples collected from fields in West Peloponnese (except WP 2 vs. WP 7). Some samples collected from greenhouses in West Greece and Peloponnese were not differentiated from the samples collected from fields: WG was not differentiated from WP 5–7 and EP, and WP 6 was not differentiated from EP. One sample from Crete (CR 2) was similar to some samples from West Peloponnese (WP 6 and WP 7), as well as the northern samples (MA 1 and NP), which were similar to each other as well (Table 5B).

There was evidence for isolation by distance in the Greek dataset (*R*_*XY*_ = 0.436, *R*^*2*^ = 0.190, *P* = 0.011), and this relationship was stronger when the three samples from Crete were removed from the analysis (*R*_*XY*_ = 0.579, *R*^*2*^ = 0.335, *P* = 0.001). There was also a strong relationship when only samples from Peloponnese were analyzed (*R*_*XY*_ = 678, *R*^*2*^ = 0.460, *P* = 0.001). In Crete, however, isolation by distance was not detected (*R*_*XY*_ = 0.963, *R*^*2*^ = 0.927, *P* = 0.186). Isolation by distance in the field samples was much greater than that between greenhouses (*R*_*XY*_ = 0.791/-0.219, *R*^*2*^ = 0.625/0.048, *P* = 0.005/0.113; fields/greenhouses).

AMOVA indicated that genetic variation was not explained by host plant families, but there was a significant percentage of variation explained by habitat (fields vs. greenhouses), and among the samples within each of these groups (Table 4). Samples from fields (all in Peloponnese) displayed significantly greater genetic diversity and less population differentiation than those from greenhouses (allelic richness: 3.380 vs. 3.139, *P* = 0.006; *Ho/H*_*E*_: 0.497/0.536 vs. 0.404/0.454, *P* < 0.001; *F*_*ST*_: 0.012 vs. 0.078, *P* = 0.003; fields vs. greenhouses, respectively). Comparison of groups of samples differing in host plant family (Table 1) did not indicate differences in F statistics, heterozygosity or allelic richness (all *P* > 0.05) (data not shown).

Analysis with GESTE revealed that a model including habitat (field vs. greenhouse) had the highest posterior probability (*Pr* = 0.407) of explaining the genetic structure. The population structure in Greece was also partially explained by longitude (*Pr* = 0.260) followed by a model which combined the habitat and longitude factors (*Pr* = 0.174).

**Figure 3:**
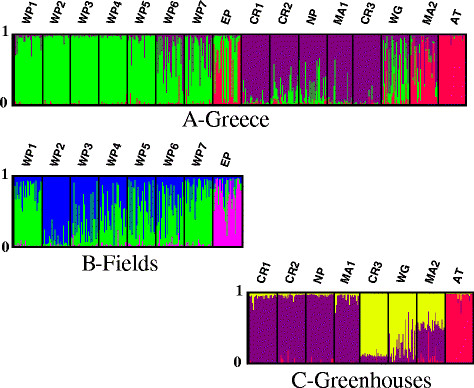
**Genetic clusters of*T. vaporariorum*sampled in Greece assigned by Bayesian analysis implemented in STRUCTURE[**[52]**].** In each graph individuals are represented by vertical bars broken into *K* differently colored genetic clusters, with length proportional to assignment in each cluster. Probability of assignment of each individual is indicated from 0 to 1. Individuals are grouped by sample with the sample code listed at the bottom of the bar graph. **A**: Analysis of all 16 samples, 474 individuals, *K* = 3. **B**: Analysis of a subset of 8 samples collected from fields, 239 individuals, *K* = 3. **C**: Analysis of a subset of 8 samples collected from greenhouses, 235 individuals, *K* = 3.

The Bayesian analysis of population structure in Greece indicated the presence of three major genetic clusters (Figure 3A). Samples from fields of West Peloponnese (WP 1–7) formed cluster 1 (*K*_*1*_). Cluster 2 (*K*_*2*_) consisted of samples from Crete and from two mainland locations (NP and MA 1), and the sample from West Greece (WG) had a mixed assignment (WG: *K*_*1*_ = 0.475 and *K*_*2*_ = 0.397). Samples AT and MA 2 formed cluster 3 (*K*_*3*_). Mixed assignments were indicated for MA 2 (*K*_*3*_ = 0.599 and *K*_*2*_ = 0.279) and EP, which grouped either with the other samples from the Peloponnese (*K*_*1*_), or with cluster 3 (*K*_*3*_) (EP: *K*_*3*_ = 0.515 and *K*_*1*_ = 0.461).

Since habitat (field vs. greenhouse) was a major component of the genetic structure indicated in our initial analysis and our analysis with GESTE, we analyzed subsets of samples from greenhouses and fields separately in STRUCTURE to investigate possible sub-structuring (Figure 3B and C). Analysis of the subset sampled from fields (*K*_*1*_ in the initial analysis) revealed the presence of three sub-clusters (*K* = 3). Majority of samples (WP 3-WP 6) were not well-resolved and assigned to both *K*_*1.1*_ and *K*_*1.2*_, however samples WP 1 and WP 7 had higher assignment probability to *K*_*1.1*_ (0.80 for WP1, and 0.825 for WP7). Sub-cluster 2 (*K*_*1.2*_) was best represented by WP 2 (with assignment probability of 0.90). Sub-cluster 3 (*K*_*1.3*_) consisted of the sample from East Peloponnese (EP: *K*_*1.3*_ = 0.754). The subset of samples from greenhouses (*K*_*2*_ and *K*_*3*_ in the initial analysis) was characterized best by three well-resolved genetic sub-clusters (*K* = 3) (Figure 3C), with 71-98% assignment rate. Sub-cluster 1 (*K*_*2.1*_) consisted of two Cretan samples (CR 1 and CR 2) and samples from Peloponnese and the mainland (NP, MA 1 and, in part, MA 2). CR 3, however, formed sub-cluster 2 (*K*_*2.2*_) with WG and MA 2. MA 2 was only partially resolved and grouped both with *K*_*2.1*_ (0.499) and *K*_*2.2*_ (0.450). Thus, populations were differentiated even within a relatively short distance on the Island of Crete. AT formed a sub-cluster 3 (*K*_*3.1*_).

## Discussion

Agricultural ecosystems can serve as temporal oases and increase distribution of many beneficial and pest species [5],[58]. The invasive pest greenhouse whitefly (*Trialeurodes vaporariorum)* has a cosmopolitan distribution [25],[26]. In this study, we examined population genetic structure among samples from its Northern (Finland) and Southern (Greece) European distribution range. There was no evidence of gene flow between the different climate zones. Dispersal of *T. vaporariorum* appears to be limited, since we found significant spatial population genetic structure among samples in both countries. Samples from Finland were less diverse and showed greater genetic differentiation than samples from Greece, which could be explained by differences in agroecosystems found in the different climate zones. In Greece, habitat (field vs. greenhouse) explained population genetic structure, but in Finland, genetic structure was dictated by host plant species. Related whiteflies in the *B. tabaci* complex also show population genetic structure [59]. However, population differentiation of *B. tabaci* (Med) in Tunisia is not related to host plant species nor is it related to type of agroecosystem in Greece [35],[60].

### The role of agroecosystems

We hypothesized that greenhouses contribute to population genetic structure in *T. vaporariorum* by limiting dispersal and gene flow among populations, and that populations in the North are more genetically differentiated than populations in the South. In Finland, our samples of *T. vaporariorum* were limited to greenhouses (since in this country this species is not able to persist year-round in agricultural fields), whereas in Greece we sampled from both greenhouses and fields. The extent of the area inhabited by homogeneous populations was approximately 1–10 km straight line distance in Finland and approximately 100 km in Greece, indicated in our analyses as samples with non-significant differences in pairwise *F*_*ST*_ (Finland: PJ 1–5, NR 1; Greece: WP 1–7), and shared cluster assignment in Bayesian analysis (Finland: PJ 1–5; Greece: WP 1–7). Whiteflies sampled at these spatial scales also showed significant isolation by distance. Although whiteflies are poor fliers, natural dispersal of up to 20 km has been observed previously for the related *B. tabaci* species [41]. Low pairwise *F*_*ST*_ values and significant isolation by distance even between remote populations in Greece suggest that the presence of agricultural fields and wild host plants year-round [61] enables greater connectivity among *T. vaporariorum* populations. Nevertheless, greenhouse agroecosystems increase genetic structure in both climate zones. Similarly, populations of whiteflies, *B. tabaci* (Med), and moths, *Trichoplusia ni,* inhabiting greenhouses in the United States and Canada, respectively, were characterized by higher genetic structure than those inhabiting fields [62],[63].

Although frequent high mortality events occur in agroecosystems in both climate zones (via chemical and biological control methods), the high abundance of less enclosed/hermetic agroecosystems (fields, plastic tunnels and greenhouses with constantly open vents) and suitable climate in the South reduces effectiveness of pest management. On the other hand, isolation of populations in more enclosed agroecosystems, such as those in the North, can create an opportunity for the development of insecticide resistance through natural selection [39]. In open environments resistance genes might be less easily fixed, but they could spread over longer distances, as indicated by the higher connectivity of field populations in our study. Populations of insect pests inhabiting greenhouses are often characterized by higher insecticide resistance than those in the fields [58].

Agroecosystems, particularly greenhouses, could affect genetic structure of pests not only by placing limitations on pest dispersal, but also by limiting their population size. Crop management practices and frequent chemical insecticide exposure can cause population bottlenecks, leading to reduction in within-population genetic variation (increases in homozygosity), as well as increase in between-population genetic differentiation [18]. Higher genetic diversity (allelic richness and heterozygosities) of Greek *T. vaporariorum* populations indicated a larger gene pool and overall population size and possibly a lower frequency of bottlenecks in the South than in the North. This poses a threat to effective pest management in the Mediterranean region. However, we observed higher than expected homozygosity in samples from both countries (reflected by deviations from HWE; Table 3). Although deviations in HWE could reflect technical problems in genotyping, they could also result from population bottlenecks and inbreeding, and homozygote excess in insect populations inhabiting agroecosystems is not uncommon [59],[64]. Nevertheless, samples deviating from HWE did not occur more frequently in the North than in the South, and in Greece, samples deviating from HWE were collected from fields as well as from greenhouses.

Despite the frequent stochastic events in agroecosystems that can reduce genetic diversity, our results indicated that *T. vaporariorum* is able to persist over years in the same greenhouses in Finland. Samples collected from the same greenhouses in 2010 and 2011 were not genetically differentiated from each other in all but one case. Prevalence of the pest year-round might eventually allow further spread into natural ecosystems. Pest persistence in agroecosystems can create propagule pressure to natural habitats and favor utilization of wild host plants that surround greenhouses and fields (Ovcarenko et al. unpubl.). Adaptation to crop species could also lead to the development of a preference for particular wild host plants with similar chemistry, as was observed for *Tetranychus urticae*[15],[65].

### The role of host plants

Occupation of agroecosystems and differences in their individual management in the two climate zones could potentially allow *T. vaporariorum* to specialize and adapt to particular host plant species or their cultivars*.* Although *T. vaporariorum* is a polyphagous insect, it is able to develop preference not only for certain plant species [66], but also for particular varieties or cultivars [67]. In Finland, major genetic clusters were characterized by the two common host plants (Figure 2). In Greece, however, host plant taxonomic family did not explain population structure. Absence of host associations in Greece may reflect the frequent alterations of host crops that are dictated by the market. On the other hand, the limited number of samples restricted to particular host plant species may have prevented detecting a possible association.

It is known that *T. vaporariorum* prefer cucumber hosts to tomato, and have higher fecundity and shorter development time on cucumber as well [28]. Thus, larger and more diverse populations would be expected on cucumber. On the contrary, our study indicated that Finnish samples from tomato were characterized by larger population size, with higher heterozygosity and allelic richness and lower values of *F* statistics than samples from cucumber. This might also be due to differences in individual management of agroecosystems. Cucumber crops are changed every three to four months, whereas tomato crops are maintained in greenhouses for nine to ten months, leading to more frequent reductions in pest population size in greenhouses growing cucumber than in those growing tomato. The result might also be a sampling effect: there were more greenhouses growing only tomato than those growing only cucumber (nine and five, respectively) (Table 1). However, our sampling reflects the tendency in Finland for more common cultivation of tomato than cucumber [68].

Associations between particular host plant species and genetic structure of *T. vaporariorum* populations might alternatively reflect different introduction sources, as well as varying frequencies of repeated introductions [69]. When there is a single introduction source, e.g. infested plant material from a single supplier, even isolated populations experiencing no gene flow will not show genetic differentiation, since all populations will share the same alleles present in the source [70]. One sample in Finland (KR) that formed a distinct cluster in our Bayesian analysis was collected from a site that has been producing its own cucumber cultivar since 2005. Although such conditions could favor adaptation of *T. vaporariorum* to this cultivar, KR is also somewhat geographically isolated from other agroecosystems and is surrounded by forests, which potentially limits insect dispersal. Therefore, it is difficult to determine if its genetic differentiation is due to adaptation or isolation. Other samples from Finland collected from cucumber (except NL) clustered together (LH, NR 2 and TJ 1–2; Figure 2C). Such genetic structure could reflect either adaptation to the host, or a common vector or origin of *T. vaporariorum*. Although growing different crops, greenhouses PJ1-5, NR1-3, NL, HR, ML, LH and TJ2 had all obtained seedlings from the same producer, and some similarities between these samples could be seen in our analysis with STRUCTURE (Figure 2A). However, origin of seedlings did not explain local *F*_*ST*_ in our analysis with GESTE, whereas the model with host plant species explained the data best. In Greece, populations from greenhouses in Crete (CR 1 and CR 2) clustered with distant locations in the mainland (NP and MA 1; Figure 3A, C), suggesting that human-mediated transfer of whiteflies has occurred between these distant locations. Human-mediated transfer of *B. tabaci* between northern and southern regions of Greece has been noted previously [32]. Unfortunately, no information on the origin of seedlings in Greece was available for our analysis.

### Limitations of the study

Our study compares pest populations living in different climate zones and subjected to different kinds of agroecosystems, so it may not be surprising that we have detected differences in population structure in the North and South. Moreover, our results might be affected by differences in the timing of sampling of *T. vaporariorum* in Finland and Greece, which matched periods of high insect abundance. In Finland maximum abundance peaks are in spring, whereas in Greece whiteflies are abundant year-round. Sampling *T. vaporariorum* in Finland in spring, when dispersal is low or non-existent might have facilitated detecting significant population structure. However, persistence of the populations over two years in the same greenhouses indicates that genetic structure is present despite the sampling period. Furthermore, no differences in population structure of *B. tabaci* in Greece were detected between late and early sampling periods from both field and greenhouse populations [35].

## Conclusions

Greenhouse agroecosystems contribute to population genetic structure in *T. vaporariorum* by limiting gene flow among populations. Populations in Finland sampled from greenhouses are less diverse and more genetically differentiated than populations in Greece, collected from both greenhouses and fields. Within Greece, pest populations inhabiting greenhouse agroecosystems were more genetically differentiated than those inhabiting fields, and habitat (field vs. greenhouse) together with longitude explained population genetic structure. In contrast, host plant species (tomato vs. cucumber) explained population genetic structure in Finland. The differing influence of type of agroecosystem and potential host plant adaptation on population genetic structure of the pest in different climate zones highlights challenges for the management of a cosmopolitan invasive pest species.

## Competing interests

The authors declare that they have no competing interests.

## Authors’ contributions

IV, IO, AT were the authors of original idea. IO, LL and IV took part in sample collection in Finland, whereas DEK and AT collected samples from Greece. NG participated in microsatellite development. DEK extracted DNA of samples from Greece, whereas IO and KEK carried all further sample and data analysis steps of all samples. KEK, NG and AT advised on data analysis methods. IO drafted the manuscript. All authors participated in preparation of the manuscript, read and approved the final manuscript.
